# A systematic review of effective quality feedback measurement tools used in clinical skills assessment

**DOI:** 10.12688/mep.18940.1

**Published:** 2022-02-25

**Authors:** Akram Alsahafi, Davina Li Xin Ling, Micheál Newell, Thomas Kropmans

**Affiliations:** 1 College of Medicine, Nursing and Health Sciences – School of Medicine, National University of Ireland, Galway, Galway, Galway. Co, H91 V4AY, Ireland; 2 Department of Medical Education, College of Medicine, Taif University, Saudi Arabia, P.O Box 11099, Taif 21944, Saudi Arabia

**Keywords:** Objective Structured Clinical Examination (OSCE), effective feedback, measurement, quality, determinant, Kappa.

## Abstract

Background:

Objective Structured Clinical Examination (OSCE) is a valid tool to assess the clinical skills of medical students. Feedback after OSCE is essential for student improvement and safe clinical practice. Many examiners do not provide helpful or insightful feedback in the text space provided after OSCE stations, which may adversely affect learning outcomes. The aim of this systematic review was to identify the best determinants for quality written feedback in the field of medicine.

Methods:

PubMed, Medline, Embase, CINHAL, Scopus, and Web of Science were searched for relevant literature up to February 2021. We included studies that described the quality of good/effective feedback in clinical skills assessment in the field of medicine. Four independent reviewers extracted determinants used to assess the quality of written feedback. The percentage agreement and kappa coefficients were calculated for each determinant. The ROBINS-I (Risk Of Bias In Non-randomized Studies of Interventions) tool was used to assess the risk of bias.

Results:

14 studies were included in this systematic review. 10 determinants were identified for assessing feedback. The determinants with the highest agreement among reviewers were specific, described gap, balanced, constructive and behavioural; with kappa values of 0.79, 0.45, 0.33, 0.33 and 0.26 respectively. All other determinants had low agreement (kappa values below 0.22) indicating that even though they have been used in the literature, they might not be applicable for good quality feedback. The risk of bias was low or moderate overall.

Conclusions:

This work suggests that good quality written feedback should be specific, balanced, and constructive in nature, and should describe the gap in student learning as well as observed behavioural actions in the exams.  Integrating these determinants in OSCE assessment will help guide and support educators for providing effective feedback for the learner.

## Introduction

During their undergraduate education, medical and health sciences students are subjected to numerous clinical practical assessments in order to evaluate their performance ^1^. Feedback is a fundamental and important learning tool in medical education ^2^. Good and effective feedback assists students in accomplishing both learning and professional development, enhancing student motivation and satisfaction ^3– 5^. The Objective Structured Clinical Examination (OSCE) is a commonly utilized clinical skills assessment in medical and health sciences that has a positive impact on medical education ^6^. OSCE is useful in the field of medicine for evaluating student performance for a variety of reasons; the OSCE will simulate the realities of clinical practice, enhancing students' confidence and ensuring safe clinical practice, with assessment based on objective determinants ^7– 10^. The OSCE is a valid and reliable assessment tool in a variety of fields, including medicine ^9, 11– 15^

During the OSCE, examiners are requested to input the students' observed marks on score sheets (without knowing total marks) and can also provide their professional opinion on students’ performance using the Global Rating Scale (GRS) (Fail, Borderline, Pass, Good, Excellent) based on experience ^16, 17^. Previous research has shown a mismatch between observed Marks and GRS ^18^. For example, the student may score a high result in the observation section, but receive a ‘fail’ for their Global Rating Score and potentially *vice versa*.

However, written feedback for OSCE is optional, despite previous research showing it to have a significant positive impact on student's learning outcomes ^19, 20^. It is argued that many examiners may find it difficult to offer detailed or useful written feedback during OSCE evaluation ^21, 22^ due to time constraints as well as a ‘judgement dilemma’ of not knowing how much feedback or the type of feedback to give ^23^.

Even if written feedback is provided, to date there is no recognised objective measurement scoring tool that measures the quality of written feedback from the OSCE. Measuring the quality of written feedback will help examiners to improve their skills in feedback delivery, as well as encourage students to understand the OCSE marks they received and where they can improve in the future. In other fields of education, feedback quality measurement tools are used effectively to improve the quality of written feedback for students ^24– 26^. In order to develop such a tool, it is necessary to identify the determinants that result in effective written feedback. The main objectives of this systematic review are to identify and evaluate studies that have measured written feedback quality.

## Methods

### Study design

This a comprehensive systematic review to identify the most relevant determinants that describe good and effective written feedback in the field of medicine. Measurement tools that measure feedback quality both quantitatively and qualitatively will be included.

### Search strategy and publication sources

CINHAL, PubMed, Medline, Embase, Scopus and Web of Science were searched for relevant studies published from January 2010 until February 2021. We used the following keywords: “OSCE “, “objective structured clinical examination*”, “medical student*”, “medical education”, “clinical skill*”, “clinical setting.” AND “formative feedback “, “constructive feedback”, “effective feedback”, “qualitative feedback”, AND “quality “, “scoring”, “measurement*/ measuring”, “assessment*/ assessing”.

We sought assistance from a university librarian to enhance our search strategy. The reference section of initially selected studies was also searched thoroughly for any additional relevant publications. A bibliographical database was created to store and manage the references.

### Selection of articles

**Figure 1.**
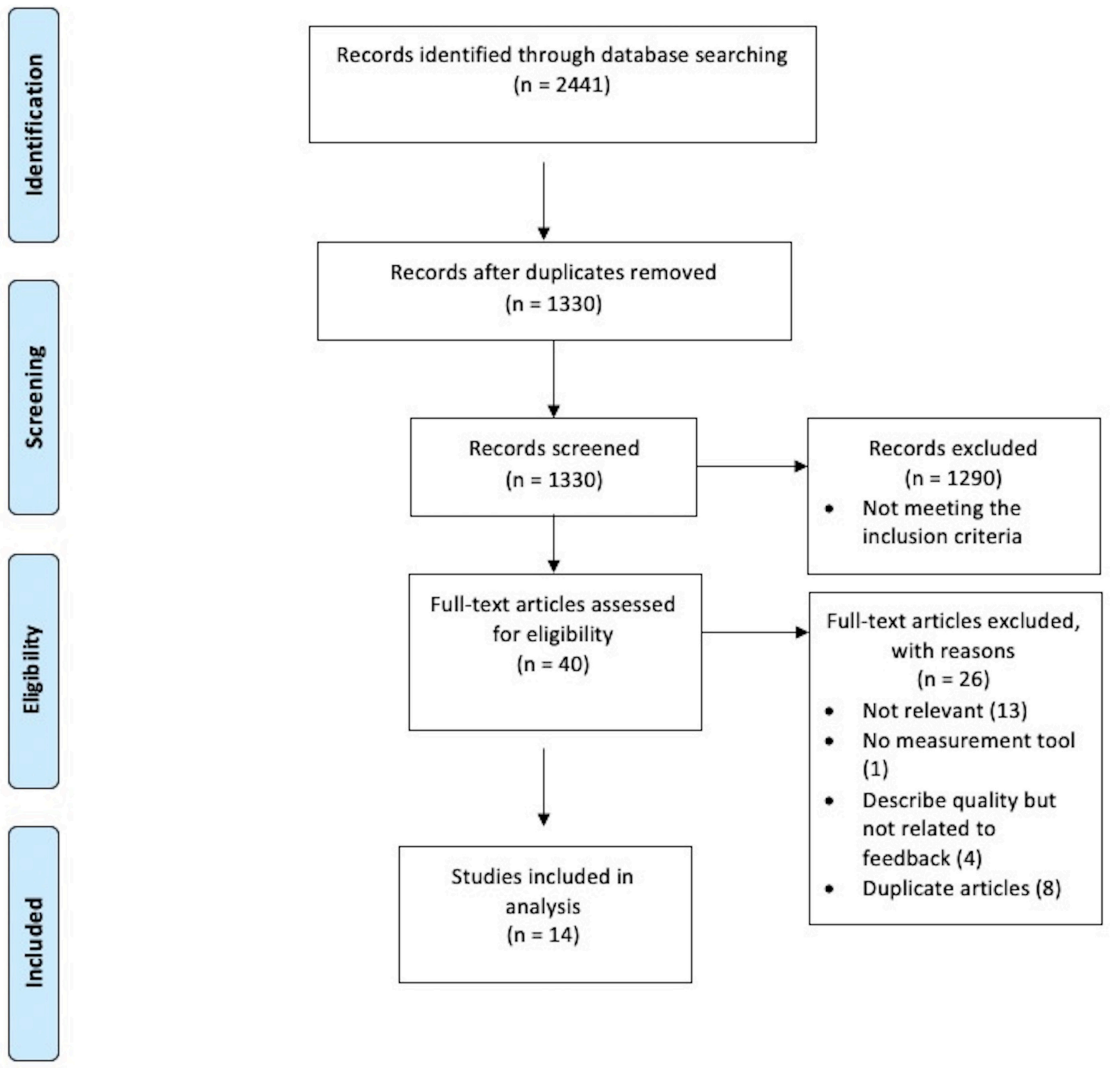
Flow diagram showing study selection process.

Each author independently screened retrieved articles against inclusion and exclusion criteria, and as a team agreed on the included studies. Following Preferred Reporting Items for Systematic Reviews and Meta-Analyses (PRISMA) guidelines ^27^, only studies written in the English language, published in the last 10 years in the field of medicine were included. Both quantitative and qualitative studies were included. Studies were included if they described the quality of good/effective feedback in clinical skills assessment, or attempted to evaluate the quality of feedback in clinical skills assessment (e.g., OSCE), or described the quality of written feedback by enumerating determinants of effective feedback involving undergraduate students and postgraduate trainees. Exclusion criteria included papers not written in English language, case reports, ‘grey literature’ (which includes conference proceeding studies) and commentaries. Due to different cognitive demands and scopes of practice, publications relating to nursing, paramedical disciplines, pharmacy, and veterinary education were excluded ( Figure 1). Reference lists of included studies were also explored to identify any additional studies.

### Data extraction

Four independent reviewers (A.A, D.L, M.N and T.K) identified and extracted the determinants used to evaluate written feedback from the included studies ( Table 1). An accumulated list of identified determinants and their respective definitions was compiled. Each reviewer then scored each of the included studies against the accumulated list of determinants. If the determinant was not mentioned by name specifically, it may have been used by its definition. A positive (+) sign indicated ‘matching with determinant definition’ while a negative (-) sign indicated ‘no match with the determinant definition’.

**Table 1.** The 10 determinants of feedback quality measurement identified.

	Determinant of feedback measurement	General description
**1**	Specific	Detailed information of what was done well or poorly.
**2**	Balanced	Contains both positive and negative comments.
**3**	Behavioural	Observed action in exam (not personal).
**4**	Timely	Given immediately after assessment is completed.
**5**	Constructive	Supportive feedback identifying a solution to area of weakness they may have.
**6**	Quantifiable	Feedback that can be used to develop detailed statistical data.
**7**	Focused	Feedback that is given around key results.
**8**	Described task	Focuses the knowledge and skills associated with a task: sufficient or insufficient.
**9**	Described gap	Detailed about what is missing in the task.
**10**	Described action plan	Detailed plan of action needed to reach one or more goals.

### Statistical analysis

Level of agreement scores (%) and Kappa coefficients between the reviewers were calculated for each determinant included. Both level of agreement and kappa were measured using an online calculator ( http://justusrandolph.net/kappa/) ^28^. Percentage agreement calculates agreement by chance which is corrected for by calculating kappa. The average Kappa coefficients were interpreted as follows: <0 indicates no agreement, 0.01–0.20 indicates slight or poor agreement, 0.21–0.40 indicates fair agreement, 0.41–0.60 indicates moderate agreement, 0.61–0.80 indicates substantial agreement, and 0.81–1.00 indicates almost perfect agreement ^29^. Determinants with the highest Kappa were identified as being most useful for providing written feedback for OSCE. In addition, included studies were assessed to identify which studies were best for measuring the quality of feedback.

### Risk of bias and certainty assessment

Two independent reviewers used the ROBINS-I (Risk Of Bias In Non-randomized Studies of Interventions) tool to assess the risk of bias of each included study ( Table 3). Confounding, selection, classification, intervention, missing data, measurement, and reporting were all checked for bias. The ROBINS-I was used to assess the certainty in the body of evidence in the context of GRADE's (Grading of Recommendations, Assessment, Development and Evaluations) approach ^30, 31^.

When there were any conflicts, the entire review team was consulted, and the disagreements were then addressed by consensus.

## Results

### Search results

The initial search yielded 2441 studies ( Figure 1). After the duplicates were removed, 1330 studies remained. 1290 studies were found to be irrelevant to the main topic after scanning the title and abstract. The 40 remaining articles were thoroughly evaluated by reading the full text. A further 26 articles ^2, 7, 21– 23, 32– 44^ were removed leaving 14 studies for inclusion in this systematic review ( Figure 1).

### Content analysis

Of the 14 studies included, 7 were conducted in the United States ^23, 45– 50^, 2 each in the United Kingdom ^51, 52^, and Canada ^53, 54^, and 1 each in the Netherlands ^55^, Switzerland ^56^, and South Africa ^19^ respectively. Half of the studies involved medical students (undergraduate setting) ^46, 48, 49, 51, 53– 55^, and the other half involved medical residents (postgraduate setting) ^19, 45, 47, 50, 52, 56, 57^. 10 of the 14 studies used a scoring system or systematic framework for evaluating the quality of written feedback ^19, 45, 48– 54, 57^, while the other 4 studies had no scoring system ^46, 47, 55, 56^. Only 2 studies were conducted in multiple institution contexts ^48, 50^, while 12 were conducted in a single institution ^19, 45– 47, 49, 51– 57^ setting.

### Determinants identified

A total of 10 determinants to assess the quality of written feedback were identified from the combined 14 studies ( Table 1).

Each reviewer then scored each of the 14 included studies against the accumulated list of determinants ( Table 2).

**Table 2.** Scoring by reviewers for determinants of quality of written feedback.

	Year	Specific	Balanced	Behavioral	Timely	constructive	Quantifiable	Focused	Describe Task	Describe gap	Describe Action plan	Overall agreement % (Studies)	Kappa
Camarata, T. *et al.* ^45^	2020	+ + + +	+ + + +	- + + -	- + + +	+ + + +	+ + + +	- - + -	- - - +	- - - +	- + + -	66.7	0.33
Tekian, A. *et al.* ^46^	2019	+ + + +	- + + +	- + + +	- - - -	- + + +	+ - + +	- - + -	- - + +	- - - -	- - - -	68.3	0.37
Tomiak. A *et al.* ^53^	2019	+ + + +	- - - -	+ + + +	+ + + +	+ + + +	- + + -	+ + + +	- + + +	- + - +	+ + - +	78.3	0.57
Page, M. *et al.* ^51^	2019	+ + + +	- - + +	- + + +	- - + +	+ + + +	+ + + +	- - + +	- + + -	- - - -	- + - -	63.3	0.27
Abraham, R. *et al.* ^19^	2019	+ + + +	- - + -	- + + +	- - + +	- + + -	+ + + +	- - + -	+ + + +	+ + + +	+ + + +	71.7	0.43
Dallaghan, G. *et al.* ^47^	2018	+ + + +	- - + -	+ + + +	- - + +	- - + +	- - + -	- - + -	- + - +	- - - -	+ + - -	58.3	0.17
Karim, A. *et al.* ^48^	2017	+ + + +	- + + +	- + + +	- - + +	- + + +	- - + +	+ - + -	- + + +	- - - -	+ + - -	53.3	0.07
Gullbas, L. *et al.* ^57^	2016	+ - + +	- + + +	+ + + +	- - + -	+ - + +	- - - +	- - + -	- - - -	- - - -	+ - - -	65.0	0.30
Junod, N. *et al.* ^56^	2016	+ + + -	+ + + +	+ + + +	- - + +	- + + +	- + + +	- - + +	- - - +	- + + -	- + + -	53.3	0.07
Nesbitt, A. *et al.* ^52^	2014	- + + +	- + - -	- - + +	- - + -	- - + +	- - - -	- + + -	+ - - +	+ + + +	+ - + -	51.7	0.03
Jackson, J. *et al.* ^49^	2014	+ + + +	+ + + +	+ + + +	- - + -	+ + + +	+ + - +	- + + +	- - - +	+ - + -	+ + - +	68.3	0.37
Gauthier, S. *et al.* ^54^	2014	+ + + +	- - - -	- - + +	- - - -	- - - -	+ + + +	- - + -	+ + + +	+ + + +	+ + + +	88.3	0.77
Pelgrim, E. *et al.* ^55^	2012	+ + + +	- + - -	- - + +	- - + +	- - + +	- + + -	- + + -	- + + +	- + + -	+ + + +	50.0	0.00
Canavan, C. *et al.* ^50^	2010	+ + + +	- + + +	+ + + +	- - + -	- - + +	- - + -	+ + + -	- + + +	- + + -	- + - -	56.7	0.13
Overall agreement % (determinants)		89.3	66.7	66.7	53.6	61.9	64.3	47.6	56	72.6	58.3		
Kappa		0.79	0.33	0.26	0.07	0.33	0.20	- 0.05	0.05	0.45	0.17	--------	------

The number of determinants identified in the individual studies ranged from 7 to 10 respectively. The determinants with the highest agreement (kappa values) among reviewers were Specific (0.79 - substantial agreement), Described gap (0.45 moderate agreement), Balanced (0.33 - fair agreement), Constructive (0.33 - fair agreement), and Behavioural (0.26 - fair agreement) respectively. All other determinants had low agreement (kappa values below 0.21 - slight or poor agreement) indicating that even though they have been used in the literature, they might not be applicable for good quality feedback. The identified determinants with highest level of agreement among reviewers were included in seven of the ten studies of which the study by Abraham *et al.* ^19^ had the highest level of agreement ^19, 28, 32, 33, 35, 38, 39^. ( Table 2).

### Risk of bias

We utilized the ROBINS-I score method to analyse bias across confounding bias, selection bias, classifications bias, intervention bias, bias due to missing data, measurement bias, and reporting bias to assess the possible risk of bias ( Table 3). Almost all the studies included had low confounding, selection, and measurement biases. The overall risk of bias was low to moderate for the included studies, which is understandable considering the nonrandomized character of the research and dependence on self-reporting measures. The remaining post-intervention biases were variable, ranging from mild to moderate.

**Table 3.** Results risk of bias assessment for individual studies using ROBINS-I methods.

Study		Pre-intervention		At- intervention	Post-intervention				Overall risk of bias
First author	Year	Bias due to confounding	Bias in selection of participants into the study	Bias in classification of interventions	Bias due to deviations from intended Interventions	Bias due to missing data	Bias in measurement of outcomes	Bias in selection of the reported result	low/ moderate/ serious/ critical
Camarata ^45^	2020	low	low	low	moderate	low	low	low	moderate
Tekian ^46^	2019	low	low	low	low	low	low	low	low
Tomiak ^53^	2019	low	low	low	moderate	low	moderate	moderate	moderate
Page ^51^	2019	low	low	low	low	low	low	low	low
Abraham ^19^	2019	low	low	low	low	low	low	low	low
Dallaghan ^47^	2018	Low	moderate	Low	low	moderate	low	moderate	moderate
Karim ^48^	2017	Low	low	Low	low	low	low	low	low
Gullbas ^57^	2016	Low	low	Low	low	low	low	low	low
Junod ^56^	2016	moderate	low	Low	moderate	low	low	low	moderate
Nesbitt ^52^	2014	low	low	Low	moderate	low	moderate	moderate	moderate
Jackson ^49^	2014	low	low	Low	low	low	Low	Low	Low
Gauthier ^54^	2014	Low	Low	Low	Low	Low	Low	Low	Low
Pelgrim ^55^	2012	Low	Low	Low	Low	moderate	Low	moderate	moderate
Canavan ^50^	2010	Low	Low	Low	moderate	moderate	moderate	moderate	moderate

### Certainty in body of evidence

In systematic reviews, the GRADE working group has created a widely-accepted approach to evaluate the certainty of a body of evidence-based on a four-level system: high, moderate, low and very low. The current GRADE strategy for a body of evidence linked to interventions starts by categorizing studies into one of two groups: randomized controlled trials (RCT) or observational studies (also non-randomized studies, or NRS). The body of evidence begins with high certainty if the relevant research is randomized trials. The body of evidence begins with a low level of certainty if the relevant study is observational ^31, 58^.

## Discussion

The aim of this systematic review was to evaluate studies that have measured the quality of written feedback for clinical exams and identify which determinants should be used to provide quality written feedback. Improving the quality of written feedback for students in the field of medicine will improve student performance.

Four independent researchers critically appraised 14 studies using 10 identified determinants. The five determinants with the highest Kappa values were: (1) Specific: tutors should include details in the comment section about what the student has done in the clinical exam (Kappa 0.79); (2) Described gap: the comment should include points about what was missing in the task (Kappa 0.45); ^59^ Balanced: the comment should include both positive and negative statements about the student's performance (Kappa 0.33); (4) Constructive feedback: the comment should identify an area of improvement and give a solution to the student (Kappa 0.33); and (5) Behavioural: the comment should include observed (not personal) action in the exam (Kappa 0.26). The other five determinants were conversely deemed to be lacking in agreement, showing some form of confusion and complexity amongst the reviewers in its ascertainment. Hence, these determinants may not be considered to be a good qualitative measurement element of feedback quality.

The number of determinants in each individual study ranged from seven to ten respectively. The five key determinants appeared in seven of the ten studies in this systematic review. It may be worthwhile to consider including these five key determinants in feedback and performance assessments.

Feedback delivery is influenced by a number of factors. One of them, according to research, is that the examiner lacks the ability to translate his observation into detailed, non-judgmental, and constructive feedback ^3, 60^. Therefore, feedback will ultimately be ambiguous and meaningless to students seeking to improve their performance ^60^.

Effective feedback tools, from the perspective of educators, should include determinants that aid in the learning process, such as helping students comprehend their subject area and providing clear guidance on how to enhance their learning. Structuring feedback by using the five identified determinants will improve alignment between GRS and observed marks. That will lead to a better understanding GRS in observed marks.

Developing a digital tool to evaluate written feedback from OSCE will help in cases where there is a discrepancy between observed marks and the Global Rating Scale result. In these cases, written feedback can be utilized in case of a pass/fail decision. This could demonstrate the significance of feedback in decision-making, as well as how written feedback is viewed as a learning tool that leads to improvements in student performance. The five identified determinants with the highest kappa values could be used as a method to quantify written feedback. Tutors and educators should be made aware of these determinants prior to the OSCE so that they provide beneficial feedback to students. Having a structured comments section could also help overcome the writing challenges tutors currently face when marking the OSCE.

Further study is needed to categorize determinants and sub classify them as part of a quantification approach. This digital measurement tool in medical education will help improve students' performance and knowledge acquisition.

This systematic review had some limitations. For example, grey literature was not included in the study and we reviewed only English studies which may mean results are not generalizable. Another limitation is the focus on written feedback in one type of clinical skills assessment (OSCE). Future research should also consider other training feedback in postgraduate training as well as undergraduate training.

## Conclusion

This work suggests that good quality written feedback should be specific, balanced, and constructive in nature, and should describe the gap in student learning as well as observed behavioural actions in the exams. Integrating these five core determinants in OSCE assessment will help guide and support educators in providing effective and actionable feedback for the learner.

## Data availability

All data underlying the results are available as part of the article and no additional source data are required.

### Reporting guidelines

Zenodo: PRISMA checklist and flow diagram for ‘A systematic review of effective quality feedback measurement tools used in clinical skills assessment. https://doi.org/10.5281/zenodo.6003404 ^61^

Data are available under the terms of the Creative Commons Attribution 4.0 International license (CC-BY 4.0). 
