# Supplementary material for: A systematic review of effective quality feedback measurement tools used in clinical skills assessment
Source: MedEdPublish (2016). 2023 Jun 19;12:11. Originally published 2022 Feb 25. [Version 2] doi: 10.12688/mep.18940.2 (PMC10331851; doi:10.12688/mep.18940.2)
Supplement: Peer review history

# Peer review: Reviewer response for version 2

**DOI:** 10.21956/mep.21103.r33777

**Published:** 2023-06-29

**Authors:** Reina Abraham

**Affiliations:** 1 Nelson Mandela School of Medicine, University of KwaZulu-Natal, Durban, KwaZulu-Natal, South Africa

I have had a look at the author’s revisions as requested and is fully satisfied. I have no more comments to add.

 I approve the article for indexing.

Are the rationale for, and objectives of, the Systematic Review clearly stated?

Yes

Are sufficient details of the methods and analysis provided to allow replication by others?

Yes

Are the conclusions drawn adequately supported by the results presented in the review?

Yes

Reviewer Expertise:

NA

I confirm that I have read this submission and believe that I have an appropriate level of expertise to confirm that it is of an acceptable scientific standard.

# Peer review: Reviewer response for version 1

**DOI:** 10.21956/mep.20297.r33428

**Published:** 2023-06-08

**Authors:** Subha Ramani

**Affiliations:** 1 Brigham and Women's Hospital, Harvard Medical School, Boston, USA

The authors have provided a comprehensive description of how they conducted their systematic review on the topic of written feedback on OSCE assessments. They make a strong case for improving the quality of written feedback and also using tools to evaluate the quality of these narratives. I agree that faculty training around writing high quality and impactful narratives would be much more useful to learners than simple ratings. Specificity of narratives would guide learners in self-reflection and formulating performance improvement plans. Appropriate methodology and data collection for the purpose of the study, followed guidelines for systematic review and reaching their conclusions.

 Only minor comments:

- use of words like insightful feedback will raise the question- insightful as perceived by whom and to whom. Perhaps actionable is more appropriate.
- 'measurement' of the quality of written feedback. The implication is that only numbers can be 'objective'. This seems contradictory when we are talking about written feedback being more meaningful than just numerical ratings
- Lastly, I would like to have seen a sentence on what this study adds to the field. Many studies on verbal feedback have included all these criteria of quality- specific, constructive, actionable etc. Just a sentence that these have been stated for verbal feedback conversations and equally important when clinical teachers are required to provide written or narrative feedback where they are not having conversations with learners.

Are the rationale for, and objectives of, the Systematic Review clearly stated?

Yes

Is the statistical analysis and its interpretation appropriate?

Yes

Are sufficient details of the methods and analysis provided to allow replication by others?

Yes

Are the conclusions drawn adequately supported by the results presented in the review?

Yes

Reviewer Expertise:

Qualitative research, Feedback, Educational theories, Mentorship

I confirm that I have read this submission and believe that I have an appropriate level of expertise to confirm that it is of an acceptable scientific standard.

# Response

**Published:** 2023-06-10

**Authors:** Akram Alsahafi

**Affiliations:** National University of Ireland, Galway, Ireland

Dear Dr Subha Ramani

 Thank you for your comments on our manuscript. Your constructive suggestions have significantly enriched our study. We are working on a revised version and will incorporate your valuable feedback. We appreciate your time, expertise, and the significant contribution you have made to our work.

 Kind regards,

 Akram

# Peer review: Reviewer response for version 1

**DOI:** 10.21956/mep.20297.r33223

**Published:** 2023-06-05

**Authors:** Reina Abraham

**Affiliations:** 1 Nelson Mandela School of Medicine, University of KwaZulu-Natal, Durban, KwaZulu-Natal, South Africa

Thank you for requesting me to review this paper: A systematic review of effective quality feedback measurement tools used in clinical skills assessment

 This was an interesting article evaluating studies that have measured the quality of written feedback in clinical exams to identify determinants for providing quality written feedback. This is significant because developing effective feedback quality measurement tools that include these determinants will improve the quality of written feedback provided by educators, thereby assisting students in their learning process to improve their clinical skills performance.

 The introduction builds a logical case and context for the problem statement. The problem statement is well articulated and the rationale for, and objectives of, the Systematic Review is clearly stated. The literature review is up to date, well integrated and critically appraised.

 The study design is stated and is suitable for a systematic review. The search strategy using the PRISMA guidelines is described. Please provide a rationale for why you are limiting your search from Jan 2010? Why not search all? The publication sources and data extraction process are sufficiently described and referenced. Measures to ensure quality of the publication sources are explained.

 The statistical analysis is quite robust, and its interpretation are reported correctly and appropriately.

 The search results are organized and easy to understand. The determinants of quality feedback is clearly indicated and appropriate. However, the way the reviewers came about scoring each of the 14 studies against the list of determinants in Table 2, as one +, two +, three +, four + or one -ve, two -ve etc is not explicitly described. Two tables and a figure are presented and agree with the text. I would suggest rephrasing the text for figure 1: Flow diagram showing study selection process based on Preferred Reporting Items for Systematic Reviews and Meta- Analyses guidelines ^27.^

 The discussion is in the same order as the result findings. The feedback quality determinants are correctly interpreted and compared with the literature. The conclusion is drawn adequately and is supported by the results presented in the systematic review. The study limitations are explained. Practical significance or theoretical implications are discussed and guidance for future studies is offered.

 Title and abstract - The title is representative of the content and breadth of the study. The title captures the importance of the study and the attention of the reader. The abstract is complete. The results in the abstract are presented in sufficient detail. The conclusion in the abstract is justified by the information in the abstract and the text. No inconsistencies between the abstract and the text.

Are the rationale for, and objectives of, the Systematic Review clearly stated?

Yes

Are sufficient details of the methods and analysis provided to allow replication by others?

Yes

Are the conclusions drawn adequately supported by the results presented in the review?

Yes

Reviewer Expertise:

NA

I confirm that I have read this submission and believe that I have an appropriate level of expertise to confirm that it is of an acceptable scientific standard, however I have significant reservations, as outlined above.

# Response

**Published:** 2023-06-10

**Authors:** Akram Alsahafi

**Affiliations:** National University of Ireland, Galway, Ireland

Dear Dr Reina Abraham

 We are grateful for your insightful feedback and recommendations on our manuscript "A Systematic Review of Effective Quality Feedback Measurement Tools Used in Clinical Skills Assessment". Your comments have been instrumental in helping us improve our work.

 In response to your query regarding our decision to limit our search to papers from January 2010 onward, our primary aim was to capture the most recent trends and developments in written feedback for clinical skills assessments. We chose this timeframe based on preliminary research indicating a significant surge in publications related to our topic of interest around 2010. Moreover, considering the resource constraints of conducting this review, we aimed to balance achieving a comprehensive search and managing our resources. We acknowledge that this rationale was not clearly stated in our paper, and we will amend the methodology section accordingly.

 Regarding scoring studies against the determinants listed in Table 2, our approach involved a panel of four reviewers who independently evaluated each study. A determinant was assigned a plus score (+) if it was explicitly addressed within the study, with multiple plus scores (++ or +++) for a stronger presence of a determinant. Conversely, an unaddressed or absent determinant was assigned a minus score (-), with multiple minus scores (-- or ---) if the absence was particularly noteworthy. This led to each study receiving an aggregate score. We will enhance the transparency of this scoring methodology in our revised manuscript.

 Lastly, to improve the clarity of the caption for Figure 1, we propose revising it to: "Flow diagram illustrating the process of study selection employed in this systematic review, guided by the Preferred Reporting Items for Systematic Reviews and Meta-Analyses (PRISMA) guidelines."

 Thank you again for your valuable input. We look forward to any further comments or suggestions you may have.

 Kind regards,

 Akram
